# Combined Action Observation and Motor Imagery Neurofeedback for Modulation of Brain Activity

**DOI:** 10.3389/fnhum.2016.00692

**Published:** 2017-01-10

**Authors:** Christopher L. Friesen, Timothy Bardouille, Heather F. Neyedli, Shaun G. Boe

**Affiliations:** ^1^Laboratory for Brain Recovery and Function, Dalhousie UniversityHalifax, NS, Canada; ^2^Department of Psychology and Neuroscience, Dalhousie UniversityHalifax, NS, Canada; ^3^Biomedical Translational Imaging Centre, IWK Health CentreHalifax, NS, Canada; ^4^School of Physiotherapy, Dalhousie UniversityHalifax, NS, Canada; ^5^School of Health and Human Performance, Dalhousie UniversityHalifax, NS, Canada

**Keywords:** neurofeedback, motor imagery, action observation, electroencephalography, brain computer interface

## Abstract

Motor imagery (MI) and action observation have proven to be efficacious adjuncts to traditional physiotherapy for enhancing motor recovery following stroke. Recently, researchers have used a combined approach called imagined imitation (II), where an individual watches a motor task being performed, while simultaneously imagining they are performing the movement. While neurofeedback (NFB) has been used extensively with MI to improve patients' ability to modulate sensorimotor activity and enhance motor recovery, the effectiveness of using NFB with II to modulate brain activity is unknown. This project tested the ability of participants to modulate sensorimotor activity during electroencephalography-based II-NFB of a complex, multi-part unilateral handshake, and whether this ability transferred to a subsequent bout of MI. Moreover, given the goal of translating findings from NFB research into practical applications, such as rehabilitation, the II-NFB system was designed with several user interface and user experience features, in an attempt to both drive user engagement and match the level of challenge to the abilities of the subjects. In particular, at easy difficulty levels the II-NFB system incentivized contralateral sensorimotor up-regulation (via event related desynchronization of the mu rhythm), while at higher difficulty levels the II-NFB system incentivized sensorimotor lateralization (i.e., both contralateral up-regulation and ipsilateral down-regulation). Thirty-two subjects, receiving real or sham NFB attended four sessions where they engaged in II-NFB training and subsequent MI. Results showed the NFB group demonstrated more bilateral sensorimotor activity during sessions 2–4 during II-NFB and subsequent MI, indicating mixed success for the implementation of this particular II-NFB system. Here we discuss our findings in the context of the design features included in the II-NFB system, highlighting limitations that should be considered in future designs.

## Introduction

Therapies involving the mental simulation of movements have drawn increased attention from researchers in the past 10 years. Such therapies have been shown to hold utility as adjuncts to use-dependent therapies in stroke rehabilitation, or as gateway therapies for patients whose limbs are too impaired to engage in traditional (i.e., movement-based) rehabilitation(Sharma et al., 2006; Wang et al., 2010; Braun et al., 2013). The two types of mental simulation therapy with the strongest claims to efficacy are motor imagery (MI) (Braun et al., 2006, 2013; Liu et al., 2014) and action observation (AO) (Garrison et al., 2013; Kim, 2014). Recently, researchers have used a combined MI/AO approach: here an individual *watches* a motor task being performed repetitively, *while simultaneously imagining* they are performing the movement themselves. This approach of “Imagined Imitation” (II) has been shown to facilitate corticospinal excitability to a greater degree than either AO or MI alone (Sakamoto et al., 2009; Ohno et al., 2011; Tsukazaki et al., 2012; Wright et al., 2014), and to increase brain activity in several regions critical for motor learning and performance over and above that seen in AO or MI (Macuga and Frey, 2012; Nedelko et al., 2012; Villiger et al., 2013; Kondo et al., 2015).

Another innovation, recently garnering much attention for its applications in neuro-prosthetics and as a supplement to the use of MI for stroke rehabilitation, is neurofeedback (NFB). Neurofeedback refers to digital interactive systems that put an individual into a control-theoretic closed feedback loop with a sensory representation of their brain activity. The individual is afforded the opportunity to “find a way” to move the system to the state explicitly defined as optimal (i.e., the win-state). Gaining this control requires the individual to create an association between the neural modulation required to elicit the system's win-state and the reward of success (Legenstein et al., 2010). Feedback is a well-established means of improving the ability to learn a wide variety of skills (Sutton and Barto, 1998; Wulf et al., 2002; Kelley and McLaughlin, 2012; Prewett et al., 2006), and NFB systems in particular are able to seamlessly combine negative feedback (i.e., the error correction that takes place in real time as the individual attempts to alter their brain activity) and positive feedback (i.e., highlighting the individuals progress through the use of reinforcing stimuli). The combination of positive and negative feedback is highly advantageous for the promotion of motor learning, as it has been shown that negative feedback enhances procedural (Wächter et al., 2009; Abe et al., 2011) and skill motor learning, while positive feedback has been shown to improve retention skills gained through motor learning (Ávila et al., 2012; Galea et al., 2015). Furthermore, the increase in interactivity inherent in the provision of feedback—in and of itself—has been shown to result in increased learner persistence (Ainley et al., 2002; Liaw and Huang, 2013; Croxton, 2014). Hence, another major advantage of using NFB for motor rehabilitation is the element of structure and interaction it brings to a task that may otherwise become boring easily. Given that the mechanism of action for MI and AO both crucially require ***repetitive*** task performance (Jeannerod, 1995), this aspect is far from trivial.

In the past 10 years, the coupling of MI with NFB has intensified the research community's interest in MI. This is undoubtedly driven by the fact that we have just entered the era of affordable, mobile EEG systems (Kranczioch et al., 2014; Zich et al., 2014)—meaning an MI-NFB interface has the potential to be far more accessible than with previous lab-based systems. MI-NFB has been shown to allow individuals to more efficiently engage the sensorimotor network (Hwang et al., 2009; Chiew et al., 2012; Bai et al., 2014; Boe et al., 2014), and to enhance the efficacy of MI as a therapeutic adjunct for stroke rehabilitation (Mihara et al., 2012). Given the ability of II to engage the sensorimotor system to a greater degree than MI or AO alone, it is possible that the use of II-NFB as an adjunct therapy in stroke rehabilitation could provide greater benefits than those demonstrated by the use of MI-NFB (Mihara et al., 2013; Ramos-Murguialday et al., 2013; Pichiorri et al., 2015). To date, II-NFB has only been attempted in one study (Kondo et al., 2015), where subjects performed short (4 s) blocks of II or MI (accompanied by a static image), then saw a binary indication of success during a rest period. While this represents an interesting and novel step forward, a real-time II-NFB system has yet to be tested; such a system would be of note as it circumvents a central limitation of current MI-neurofeedback systems: the fact that imagery is best accomplished with the eyes closed limits designers, making the delivery of visual, real-time feedback suboptimal. Making it ***advantageous*** to perform imagery with the eyes open, by combining MI and AO, opens up many possibilities with respect to interface design, yet to date no real-time II-NFB systems have been created.

In addition to the ability to modulate neural function with increased precision (i.e., through NFB learning), another important aspect of NFB is transfer—i.e., when the enhanced ability to control an aspect of brain function learned through NFB generalizes to a situation where NFB is not being provided. NFB learning has been shown to induce task-related changes in both white and gray matter volume, (Ghaziri et al., 2013; Butz et al., 2014) suggesting these transient changes can lead to lasting effects on the behavior of various functional neural networks. The presence of lasting changes in functional neural activity following training is referred to as NFB transfer, and it has been shown to last 6 months (Leins et al., 2007), 2 years (Gani, 2009), or even 9 years (Strehl, 2014) following NFB training. When developing NFB systems for rehabilitation from stroke, the presence of NFB transfer is a key metric, as it indicates that subsequent MI performed without NFB will be more effective than if the individual had not undergone NFB training.

To investigate the effectiveness of using NFB during II to modulate brain activity, and to test the transfer of NFB learning to subsequent MI, we created and tested an II-NFB system. Sensorimotor activity during II was quantified via desynchronization of the mu rhythm. The mu rhythm is thought to reflect a class of components that differ slightly in their location and relationship to sensorimotor processing (Pfurtscheller and Berghold, 1989), and mu desynchronization during movement, imagery, and action observation has been robustly demonstrated (Arroyo et al., 1993; McFarland et al., 2000; Muthukumaraswamy et al., 2004). This system allowed users to watch first-person videos of a complex handshake (see Supplemental Video 1 for a video depicting the task), while imagining that they themselves were executing the handshake, to receive real-time feedback regarding the quality of their II. “Quality” referred to the ability to induce sensorimotor laterality—to both up-regulate contralateral sensorimotor activity, and down-regulate ipsilateral sensorimotor activity. This pattern of activity has been shown to correlate with a re-balancing of the maladaptive interhemispheric inhibition that has been associated with more complete motor recovery from stroke (Ferbert et al., 1992; Ward et al., 2003; Dimyan et al., 2014).

Given the translational nature of NFB research, the current system was created with an emphasis on user experience (UX). We endeavored to create an interactive digital system that would engage users and spur motivation to improve throughout long experimental sessions. Feedback was provided in the form of varying video color—the videos started black-and-white, and turned to color on the basis of the electroencephalography (EEG) data being collected over the subjects' sensorimotor cortices. To optimize the onboarding of subjects, the II-NFB system contained titrated difficulty, and communicated subjects' performance to them during the experimental session. At easy difficulty levels the II-NFB system incentivized contralateral sensorimotor up-regulation, and at higher difficulty levels it incentivized both contralateral up-regulation, as well as ipsilateral down-regulation. While this experimental design means the exact nature of the feedback was not standardized between sessions, the decision to align the level of challenge presented to subjects with their aptitude was made to optimize individual learning. Moreover, this approach is consistent with best practice in rehabilitation, where tailoring the level of challenge of an intervention to each individual is common practice (Page et al., 2013).

Here we report preliminary findings related to the ability of users to modulate their brain activity as a result of engaging with the II-NFB system. Our results have implications for the future design and application of NFB systems that attempt to augment motor simulation for neurorehabilitation.

## Methods

### Subjects

Thirty-two right-handed (Oldfield, 1971), non-disabled adults (10 males; 23.7 ± 3.4 years) agreed to participate. All subjects had normal or corrected-to-normal vision, were free of neurological and movement disorders and each provided written, informed consent. Subjects were assigned to either the NFB (*n* = 17) or sham feedback (*n* = 15) group based on a pre-determined recruiting schedule to ensure that each member of the sham feedback group would have a unique member of the neurofeedback group to be yoked to (yoking is described in detail below). Experimenters were not blinded, but were given a structured script to ensure they responded in a consistent manner to any questions asked about how the NFB system worked, or for advice on how to optimize their performance. The study was conducted with approval from the Research Ethics Board at the IWK Health Centre.

### Experimental task/paradigm

**Figure 1:**
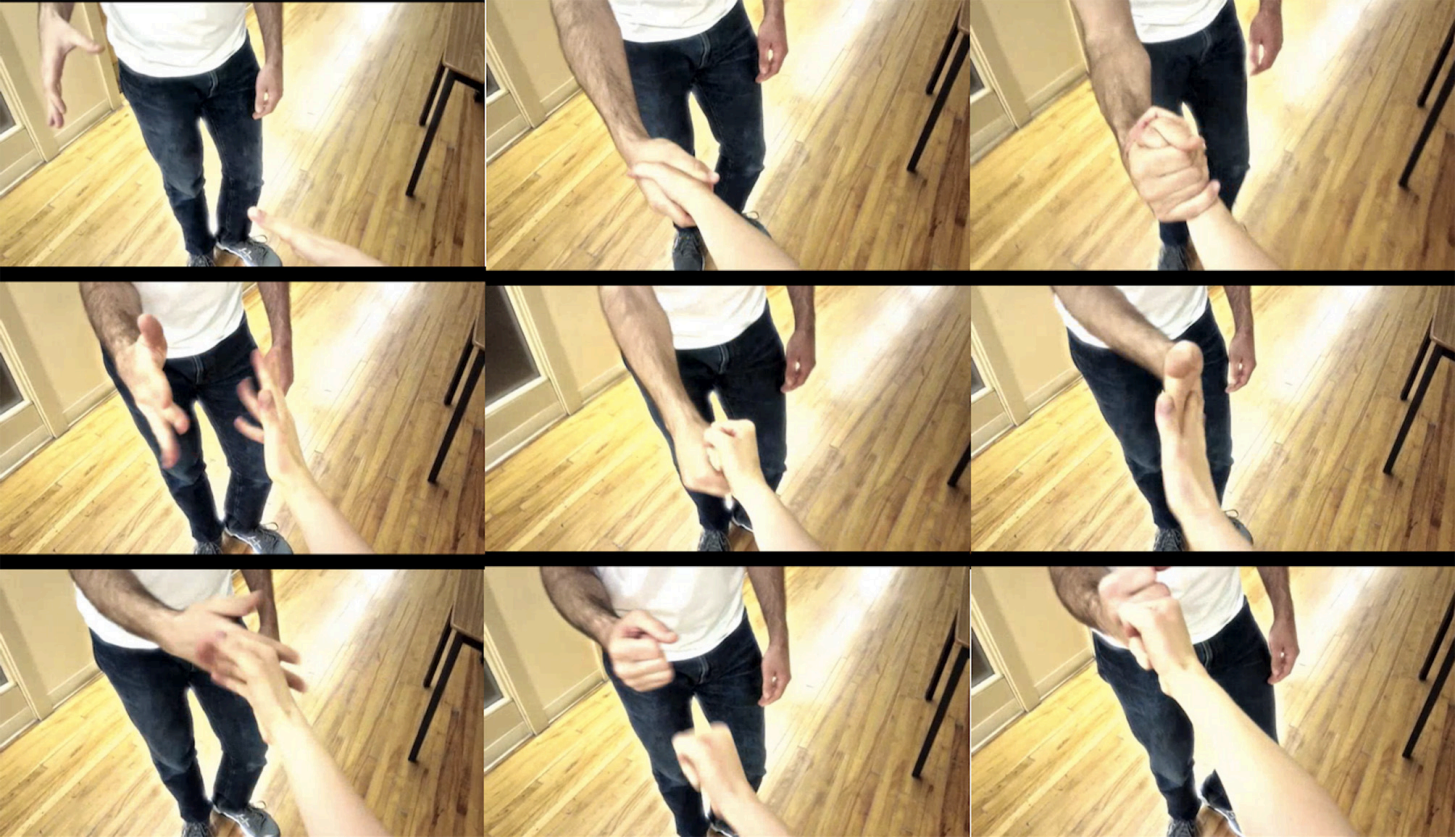
**Illustration of the complex handshake video used in the II-NFB system**.

**Figure 2:**
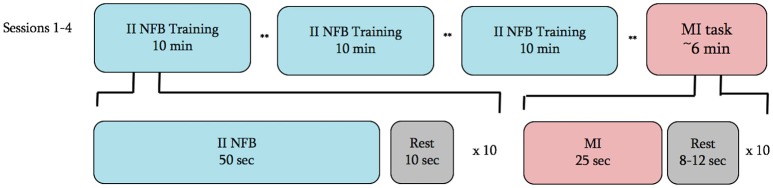
**Experimental timeline for all 4 sessions**. ^**^ = junction where the subject was asked to take as much time as they would like to rest and prepare for the next block.

Subjects in both groups were to attend four experimental sessions performed at approximately the same time of day within a 7-day period. At the beginning of the first session subjects completed the Kinesthetic and Visual Imagery Questionnaire (Malouin et al., 2007) (KVIQ) and the Edinburgh Handedness Inventory (Oldfield, 1971) to confirm ability to perform MI and hand dominance, respectively. Following completion of these questionnaires, subjects watched a 2-min video describing the NFB task, which included 2 replays of a 7-s video of a complex handshake (see Figure 1). Following the introduction video in session 1, and at the outset of sessions 2–4, subjects were prepared for EEG and EMG recording. On all study days, subjects performed 3 blocks of II-NFB training, and to test for NFB transfer, a single block of MI without NFB (Figure 2).

Each II-NFB block consisted of 10 trials (see below for description of the task being performed), with each trial lasting 50 s followed by 10 s of rest. The MI block performed at the conclusion of the II-NFB blocks consisted of 10 trials, with each trial consisting of 20 s of eyes-closed MI, followed by a rest period, the length of which was randomized on a trial by trial basis (between 8 and 12 s) in order to minimize anticipatory responses prior to the “go” cue. In the MI block, subjects were instructed to imagine they were performing the handshake from the NFB condition (Figure 1), from the same perspective and at the same speed it was presented in the II-NFB video.

### Data acquisition

The EEG signal was detected using a QuikCap (Compumedics Neuroscan, Charlotte, NC) attached to a Synamps RT system (Compumedics Neuroscan, Charlotte, NC) at a sampling rate of 1000 Hz and a band-pass of DC-333 Hz. Impedances for all electrodes was maintained at <15 kΩ throughout the experiment. Sensors C3/CP3 and C4/CP4 were used to detect activity at the left and right sensorimotor cortex, respectively. The selection of these four sensors is informed by multiple EEG studies of motor execution and imagery (Neuper et al., 2005; Pfurtscheller et al., 2006; Höller et al., 2013; Higashi and Tanaka, 2011). The electrooculogram (EOG) was obtained using self-adhering ring electrodes placed above and below the left eye, and just lateral to the left and right eye. Self-adhering ring electrodes placed overlying the left and right mastoid process served as reference. Activity of the extensor (i.e., extensor carpi radialis longus) and flexor (i.e., flexor carpi radialis) muscles of the wrists was acquired throughout using self-adhering electrodes (3 x 3 cm; Kendall-LTP, Chicopee, MA) arranged in a bi-polar configuration (inter-electrode distance of 2 cm) using the EEG electronics as described above. The ground electrode on the 128-channel QuikCap (located between AFz and Fz) was used as a ground.

### Online processing

Acquisition of the EEG and EMG data was performed in Curry 7 (Compumedics Neuroscan, Charlotte, NC). The following procedures were applied online to the continuous EEG data: re-referencing to the bilateral mastoid electrodes; high- and low-pass filters at 1 and 100 Hz, respectively; a notch filter at 60 Hz; and baseline correction (using the first 3 s of data acquired). Artifact reduction was also performed online via principal component analysis (PCA) as implemented in Curry 7, using a threshold of ± 360 mV at both vertical and horizontal occular electrodes to identify eye blinks and movements, attenuating the first component within a window of −200 to 500 ms relative to the peak of the detected artifact.

### Calculation of neurofeedback metric

Following preprocessing, 500 ms data segments were passed from Curry to MATLAB (MATLAB 8.03, The MathWorks Inc., Natick, MA, 2014) for analysis using a custom script. Task related decreases, or event related desynchronization (ERD) of the mu rhythm (the central rhythm in a 7.5–14.5 Hz window) was used to quantify sensorimotor activity. The custom MATLAB script continuously estimated power in the mu rhythm; measured via a fast Fourier transform) relative to baseline. Baseline was the mean mu power during a 15 s block obtained immediately prior to the first II- NFB block. During this 15 s block the subject silently counted backwards from 100 to 3 s, while staring at a fixation cross and keeping their arms as relaxed as possible. A single, fixed baseline (i.e., the 15 s block) was required in order to titrate the difficulty of the NFB system. A log2 function was applied to the mu power during II-NFB divided by the baseline power, producing a negative integer for all ERD segments, and a positive integer for all event-related synchronization (ERS) segments. A running average of the most recent 6 data segments (i.e., 3 s in total) was used as the metric of current mu power relative to baseline. A running average of the previous 3 s of mu power change was used in order to present the modulation of mu power in a smooth way to the subject.

In order to incentivize contralateral ERD and ipsilateral ERS during the unilateral right-handed task, a series of calculations were applied to the average mu power of each hemisphere's sensors (Equation 1), resulting in a single *NFB Score*. If the NFB Score was a positive integer, it meant they were producing contralateral ERD and ipsilateral ERS at levels above chance, and vice versa for a negative integer.

$$\begin{array}{l} \text{NFB Score}=\text{ rw log}2\left(\frac{\text{C}4\text{nfb}}{\text{C}4\text{b}}+\frac{\text{CP}4\text{nfb}}{\text{CP}4\text{b}}\right)\\ -\text{lw log}2\left(\frac{\text{C}3\text{nfb}}{\text{C}3\text{b}}+\;\frac{\text{CP}3\text{nfb}}{\text{CP}3\text{b}}\right) \end{array}$$

Equation 1. Calculations executed on average mu power, to create a singular metric that incentivizes left hemisphere decreases and right hemisphere increases in mu power, weighting each hemisphere's contribution to the score based on the difficulty level of the II-NFB system. C4nfb = mu power at sensor C4 during NFB; C4b = mu power at sensor C4 at rest; lw & rw = weighting value's for the left and right hemispheres, respectively. See Table 1 for a full list of hemisphere weighting-difficulty level pairings.

**Table 1:** **Listed are the left and right hemisphere weighting values (lw and rw from Equation 1) for each difficulty level, as well as the NFB Score thresholds for each difficulty level**.

**Difficulty Level**	**lw**	**rw**	**NFB Score Thresholds**
−1	1	0	[−8 −4]
0	1	0	[−6 −2]
1	1	0	[−4 0]
2	1	0	[−2 2]
3	1	0	[0 4]
4	1	0	[2 6]
5	1	0.1	[2 6]
6	1	0.2	[2 6]
7	1	0.3	[2 6]
8	1	0.4	[2 6]
9	1	0.5	[2 6]
10	1	0.6	[2 6]
11	1	0.7	[2 6]
12	1	0.8	[2 6]
13	1	0.9	[2 6]
14	1	1	[2 6]
15	1	1	[+2 +2]

*NFB scores above the lower threshold would move the Video Score one increment closer to black and white, while NFB scores above the higher threshold would move the Video Score one increment closer to full color saturation. For difficulty levels >14 the NFB Score threshold values increase by two ad nauseam*.

One important aspect of the NFB Score calculation was the weighting applied to each hemisphere (Table 1), which was determined by the difficulty level the subject was currently at. At low difficulty-levels (1–4) the value for the ipsilateral (right) hemisphere was not factored into the NFB Score, however the threshold values determining the video color-level increased. Conversely, at each medium difficulty-level (5–14) the ipsilateral hemisphere's weighting factor (value *rw* in Equation 1) was increased by 10%. At high difficulty-levels (>14) ipsilateral and contralateral II-NFB metrics contributed equally to the final II-NFB metric (i.e., *lw* = *rw)*, with the thresholds determining the changes in color-level increasing with each difficulty level.

### Neurofeedback system

The II-NFB system consisted of Presentation® (Version 16.05.09, www.neurobs.com) code designed to repetitively loop the video of the complex handshake (Figure 1). The color of each frame depended on a value (Video Score) passed from MATLAB to Presentation every 500 ms. The Video Score ranged from 1 to 6, corresponding to a range from black-and-white to full color saturation. At the beginning of each NFB trial, the default Video Score value was 1, meaning at the beginning of each II-NFB block the video started black-and-white.

**Figure 3:**
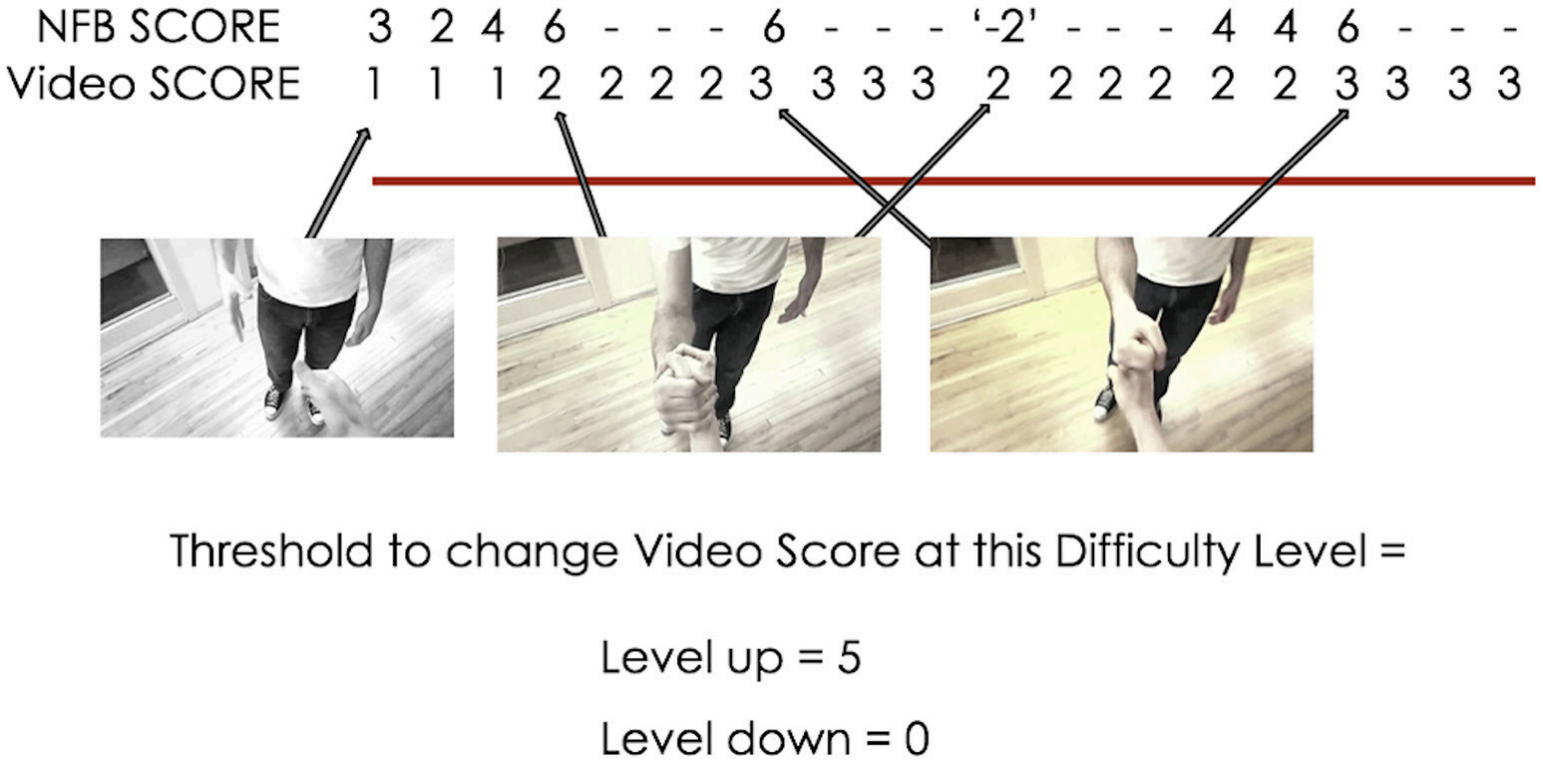
**Example of the interaction between NFB score and the video's color during II-NFB (with a new NFB score calculated every 500 ms)**. When the NFB score exceeds the lower or upper bounds of the threshold (i.e., the definition of the win and lose state at the current difficulty level), the Video Score decreases or increases by 1 (unless already at 1, then it remains at 1), and the Video Score will then not change for the next 2 s, to ensure that changes in the color gradient do not happen so quickly as to become imperceptible.

The Video Score value at each 500 ms instance was determined by comparing the current NFB Score (Equation 1) to the NFB Score thresholds for the current difficulty level (see Figure 3 for illustration; see Table 1 for a list of difficulty level-NFB Score threshold pairings). The Video Score only moved up or down one increment at a time, and each time the Video Score changed, it was not able to change during the next 2 s. These design choices were made to ensure that the changes in the color gradient (i.e., the real-time representation of NFB performance) were a smooth, intuitive and easily perceptible representation of NFB performance.

At the beginning of each experimental day, subjects began at difficulty level 1. If subjects remained at difficulty level 1 for three trials in a row, there were two difficulty levels below one (0 and −1; where the thresholds required to increase the Video Score were lowered considerably from difficulty level 1), in order to prevent any participants' initial level of competence from precluding them from progression on the II-NFB system. Upon completion of each NFB trial, the Video Score values from the last 20 s were averaged, and this value determined whether the difficulty-level increased (average color-level >4) decreased (average color-level <2), or stayed the same (average color-level 2–4).

During each rest period, a line graph depicting the difficulty level achieved by the subject throughout the day's NFB blocks was presented (Supplemental Figure 1). In conjunction with the presentation of the line graph, a happy or sad sound played if the difficulty level moved up or down, respectively. This feature was included to increase the interactivity of the system and increase subjects engagement with the task, as previous research has shown that engaging the auditory system through reward processing can increase task performance (Weis et al., 2013a,b).

Upon completion of each II-NFB session, a screen appeared thanking the subject for their effort, and stating the average difficulty level they achieved.

In addition, the difficulty levels the sham subjects would have achieved throughout the entire session were calculated by the MATLAB script, and saved to file upon completion of the experimental day. Video score and the corresponding color-level for each video frame were also saved to file to enable the provision of sham NFB.

To ensure the ERD/ERS values used to generate the NFB signal were not the result of overt movement, online analysis of the EMG signal was performed. Specifically, every 500 ms the amplitude of the full-wave rectified EMG signal from the flexor and extensor musculature of the right arm was compared to the corresponding average obtained during the baseline period (i.e., the 15 s block obtained immediately prior to the first II- NFB block). In the real-time II-NFB system, if the amplitude of the current 500 ms sample of EMG was 2 SD greater than the baseline amplitude values, EMG activity was considered excessive, and the Video Score was reset to 1. For subjects in the sham group, online EMG did not affect the Video Scores presented to them.

### Provision of sham neurofeedback

All individuals in the sham group were yoked to an individual in the NFB group. The Video Score and Difficulty levels experienced by the sham subjects during their 4 sessions were identical to those of the NFB subject they were yoked to. The MATLAB script for the Sham group accessed the text files containing the Video Score and difficulty level values for NFB subject and session the current Sham session was yoked to, and referenced these files rather than the online calculation of mu power when communicating with Presentation.

### Offline data analysis

Bilateral mastoid re-referencing, high-pass filter at 0.5 Hz, a notch filter at 60 Hz, and baseline correction (using the first 3 s of data acquired) were applied to all continuous data files. A PCA was also performed, using a threshold of ± 200 mV at both vertical and horizontal ocular electrodes to identify eye blinks and movements, with the first component in the time window −200–500 ms relative to the peak of the artifact being removed.

Pre-processed continuous EEG data were then segmented into epochs synchronized to event markers placed in the continuous data file by the II-NFB Presentation script (with unique event markers identifying the beginning and end of each block). For each session, there were 30 × 50 s epochs of II-NFB, and 30 × 10 s epochs of rest; and for the MI condition there were 10 × 25 s epochs of MI, and 10 × 8 s epochs of rest. All epochs from the NFB and MI task were concatenated into two data files, and these new files (one for each task, session, and subject) were exported to MATLAB for subsequent analysis. Consistent with the online approach described above, EMG from both real and sham NFB groups were evaluated for the presence of EMG activity in the right arm. Specifically, 500 ms data segments from the II-NFB blocks where the EMG signal from the flexor and extensor musculature of the right arm was >2 SD from the baseline period were discarded from subsequent analysis. The power in the mu rhythm was calculated (using a fast Fourier transform) in 500 ms segments, and the power at each segment was divided by a mean baseline mu power value. For the NFB task the last 4 s from the baseline calculation period (where the subject counted backwards by 3 s while remaining still) was used, for the MI task the last 4 s from the initial rest period (where the subject had their eyes closed and had been instructed to relax and remain still). The ERD/ERS values from each 500 ms segment for each task, respectively, were concatenated with the group and session independent variables, and the resulting matrices were then exported to RStudio (2015) for analysis.

### Statistical analysis

We used conditional inference random forest modeling (Breiman, 2001; Hothorn et al., 2006) (CForest) to investigate the differences in activity between the NFB and sham groups. CForest is a recursive machine learning algorithm, well-suited to modeling data with a non-normal distribution (Grandvalet, 2004; Strobl et al., 2009). This method is advantageous for the study of longitudinal NFB data, given the variability in the types of effects found in the NFB literature. CForest (1) randomly selects a subset of a full data set (bootstrap aggregation or *bagging* Breiman, 1996, 2001; Strobl et al., 2009), (2) randomly selects an independent variable (termed, variable pre-selection), and uses a permutation testing method (Strasser and Weber, 1999) to detect the split of the independent variable that renders the smallest *p*-value. The data is split along this dimension of the independent variable, resulting in two new subsets of data that are each tested using another randomly selected independent variable. The process continues until the “best split point” of a variable renders a *p* < 0.05 (Bonferroni corrected for multiple comparisons). The conclusion of this process produces a single decision tree. After a pre-selected number of trees have been grown, they are averaged, (Oliver and Hand, 1996, 2014) resulting in a single predictive model where the relationship between the independent and dependent variables can be explored in an a priori manner.

In keeping with best practices, (Tremblay and Newman, 2016) 2500 CForest decision trees were grown (Hothorn et al., 2015), using bags (i.e., initial partitions) encompassing 23.3% of the entire dataset, and testing each node with 1 randomly selected independent variable. The dependent variable in the model was event-related mu power with respect to baseline, and the independent variables of interest were group (NFB or sham) and session.

## Results

### Questionnaire score and EMG rejection

The visual and kinesthetic scores on the KVIQ were within a normal range for both groups (for the NFB group: 19.29 ± 2.85 for visual, and 19.88 ± 3.18 for kinesthetic; for the Sham group: 20.53 ± 3.42 for visual, and 20.8 ± 3.99 for kinesthetic Malouin et al., 2007). The NFB and Sham groups consisted of 17 and 15 subjects, respectively, however, two subjects in the NFB group (included in the analysis) only completed 2 sessions. Across all trials, 31.9% of the EEG data from the NFB group, and 36.9% from the Sham group were discarded due to excess EMG activity in the right flexor and extensor muscles of the digits.

### Cforest analysis of II-NFB's effect on sensorimotor activity

**Figure 4:**
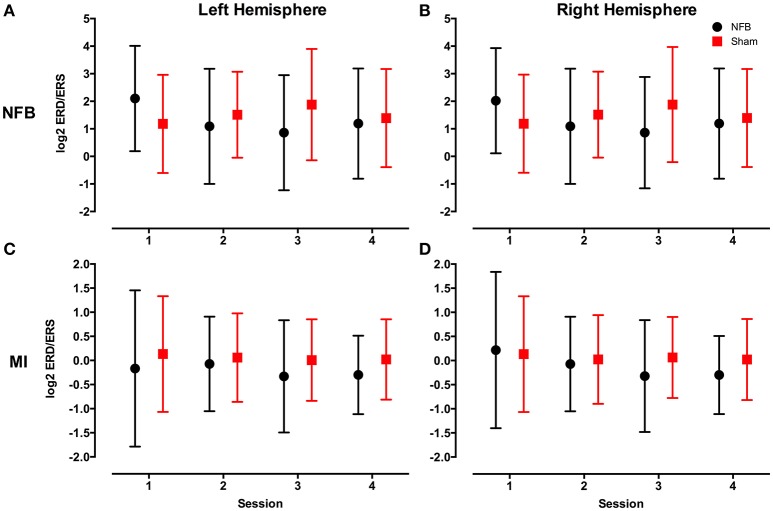
**Mean log2 mu rhythm ERD/S values from both the left (A)** and right **(B)** hemisphere during the NFB task, and left **(C)** and right **(D)** hemisphere during the MI task for the NFB (black circles) and Sham (red squares) groups. The log2 of the ERD/S values in the mu rhythm have an inverse relationship to sensorimotor activity. Bars represent standard deviation.

Figure 4 shows the distribution of ERD/S values across all sessions for the NFB and sham groups for both the NFB and MI tasks.

To investigate whether subjects in the NFB group produced more contralateral and less ipsilateral sensorimotor activity than the sham group in the II-NFB task, the significant partitions of the data, as defined by the CForest procedure, were explored in order to determine the effect of group (NFB vs. Sham) and session (1–4) on the activity of the contralateral (left) and ipsilateral (right) mu ERD/S data, respectively.

**Figure 5:**
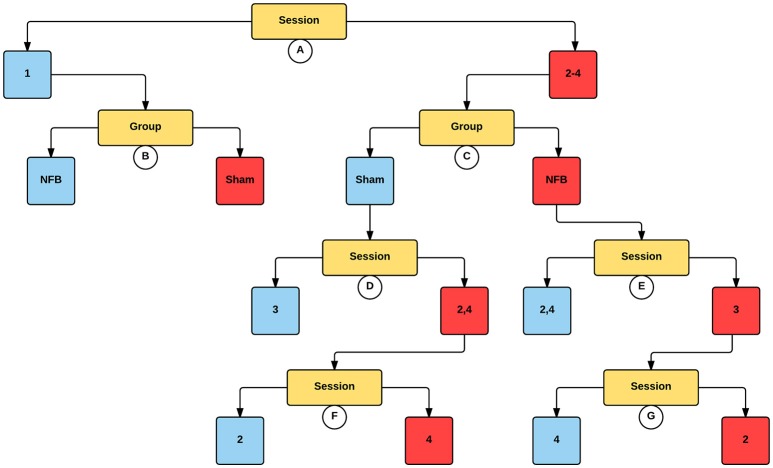
**Decision tree for the CForest predictive model of the contralateral (left) EEG sensors during the NFB task**. The data is partitioned according to split of the independent variable (either session or group) that garners the most significant effect. Where no split garners an effect of *p* < 0.05 (Bonferroni corrected) no split is shown. Red squares represent the partition with significantly lower ERD/S values (i.e., less sensorimotor activity) while blue squares represent the corresponding partition with higher ERD/S values. Split A: effect of session, with sessions 2–4 showing more sensorimotor activity than session 1. Split B: effect of group, with sham showing more sensorimotor activity than NFB during session 1. Split C: effect of group, with NFB showing more sensorimotor activity than sham during sessions 2–4. Split D: effect of session, with sessions 2 and 4 showing more sensorimotor activity than session 3. Split E: effect of session, with session 3 showing more sensorimotor activity than sessions 2 and 4. Split F: effect of session, with session 4 showing more sensorimotor activity than session 2. Split G: effect of session, with session 2 showing more sensorimotor activity than session 4.

Figure 5 shows the significant partitions of the final predictive CForest model for the contralateral hemisphere during the NFB task, explored to determine the effect of group (NFB/sham) and session (1–4) on log2 mu ERD/S values. The results of the CForest analysis showed that the largest effect was associated with the difference between sessions 1 and sessions 2–4, with sessions 2–4 having significantly lower log2 ERD/S values than session 1. While in the session 1 partition the sham group had significantly lower log2 ERD/S values than the NFB group, in accordance with our hypotheses this is reversed in sessions 2–4 (see Figure 5).

**Figure 6:**
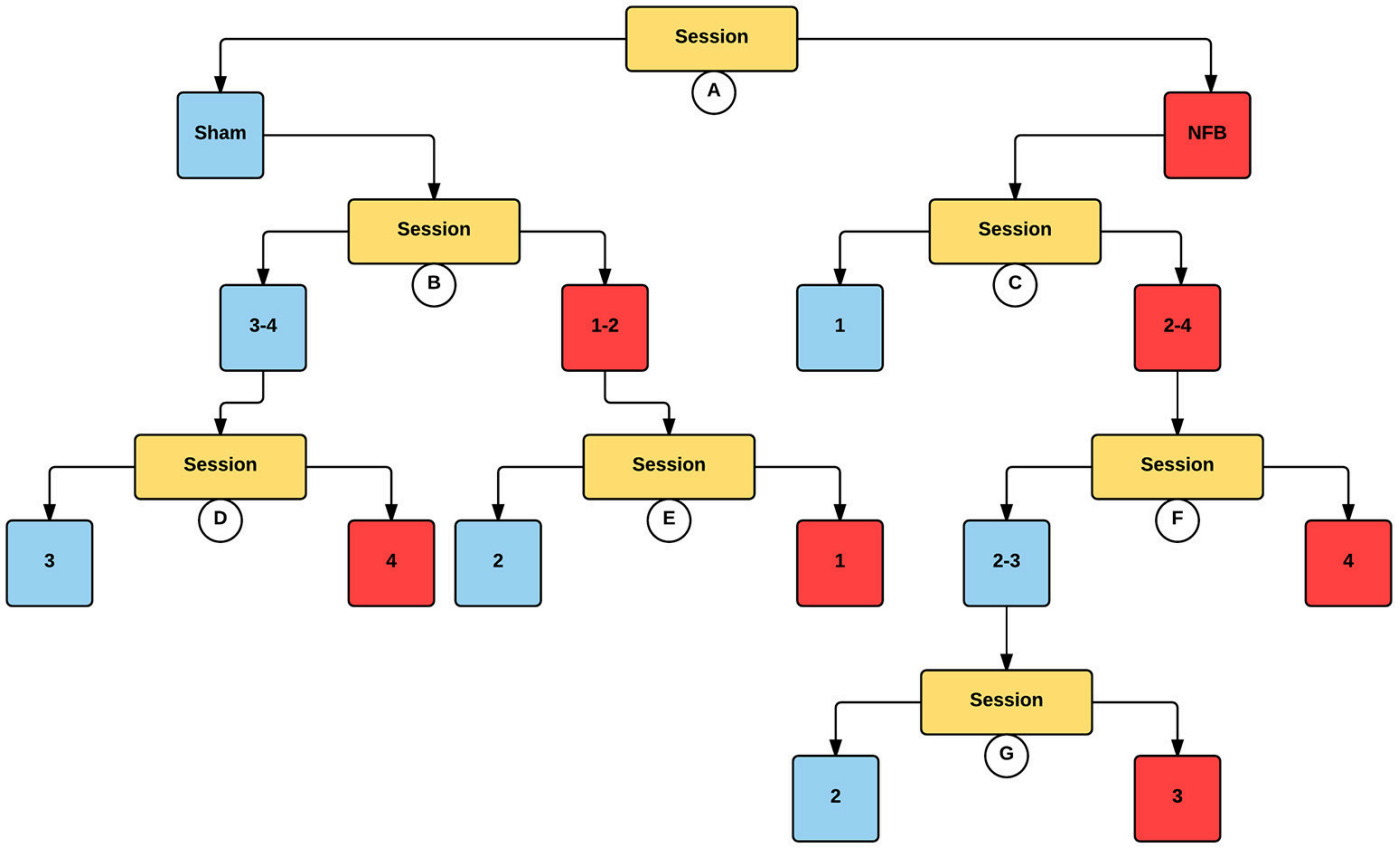
**Decision tree for the CForest predictive model of the ipsilateral (right) EEG sensors during the NFB task**. The data is partitioned according to split of the independent variable (either session or group) that garners the most significant effect. Where no split garners an effect of *p* < 0.05 (Bonferroni corrected) no split is shown. Red squares represent the partition with significantly lower ERD/S values (i.e., less sensorimotor activity) while blue squares represent the corresponding partition with higher ERD/S values. Split A: effect of group, with NFB showing more sensorimotor activity than sham. Split B: effect of session, with sessions 1–2 showing more sensorimotor activity than sessions 3–4 in the sham group. Split C: effect of session, with sessions 2–4 showing more sensorimotor activity than session 1 in the NFB group. Split D: effect of session, with session 4 showing more sensorimotor activity than session 3. Split E: effect of session, with session 1 showing more sensorimotor activity than session 2. Split F: effect of session, with session 4 showing more sensorimotor activity than sessions 2–3. Split G: effect of session, with session 3 showing more sensorimotor activity than session 2.

Figure 6 contains an analogous decision tree for the ipsilateral hemisphere during the NFB task. Results show that the NFB group has significantly lower log2 ERD/S values compared to the sham group, with this split occurring at the first level of the decision tree. While the direction of this effect aligns with the design of the NFB system in the contralateral hemisphere, finding this pattern in the ipsilateral hemisphere is the inverse of the modulation the NFB system was designed to facilitate. Furthermore, while the sham group showed lower log2 ERD/S values for sessions 1–2 compared to sessions 3–4, the NFB group had lower log2 ERD/S values in sessions 2–4 compared with session 1. Thus it appears that the sham group shows the ipsilateral effect we hypothesized to see in the NFB group.

**Figure 7:**
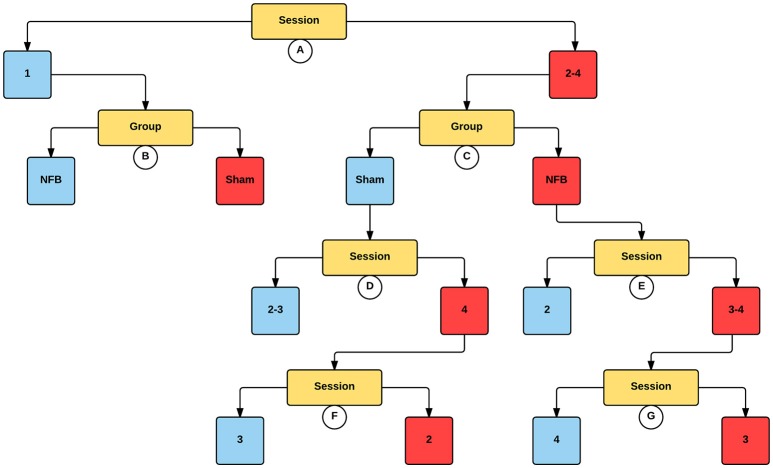
**Decision tree for the CForest predictive model of the contralateral (left) EEG sensors during the MI task**. The data is partitioned according to split of the independent variable (either session or group) that garners the most significant effect. Where no split garners an effect of *p* < 0.05 (Bonferroni corrected) no split is shown. Red squares represent the partition with significantly lower ERD/S values (i.e., less sensorimotor activity) while blue squares represent the corresponding partition with higher ERD/S values. Split A: effect of session, with sessions 2–4 showing more sensorimotor activity than session 1. Split B: effect of group, with sham showing more sensorimotor activity than NFB during session 1. Split C: effect of group, with NFB showing more sensorimotor activity than sham during sessions 2–4. Split D: effect of session, with sessions 2 and 3 showing more sensorimotor activity than session 4. Split E: effect of session, with sessions 3–4 showing more sensorimotor activity than sessions 2 and 4. Split F: effect of session, with session 2 showing more sensorimotor activity than session 3. Split G: effect of session, with session 3 showing more sensorimotor activity than session 4.

The decision tree for the mu ERD/S in the contralateral hemisphere for the MI task (Figure 7) is similar to that found for the contralateral sensors in the NFB task. Like the NFB task, the first split segregates sessions 1 from sessions 2–4, with lower log2 ERD/S values in sessions 2–4. Also mirroring the NFB results, session 1 is split by group, with the sham group showing lower log2 ERD/S values than the NFB group, while within sessions 2–4 the NFB group had lower log2 ERD/S values compared with sham. Results for the ipsilateral hemisphere during the MI task showed a similar group by session effect as the contralateral hemisphere (Supplemental Figure 2).

**Figure 8:**
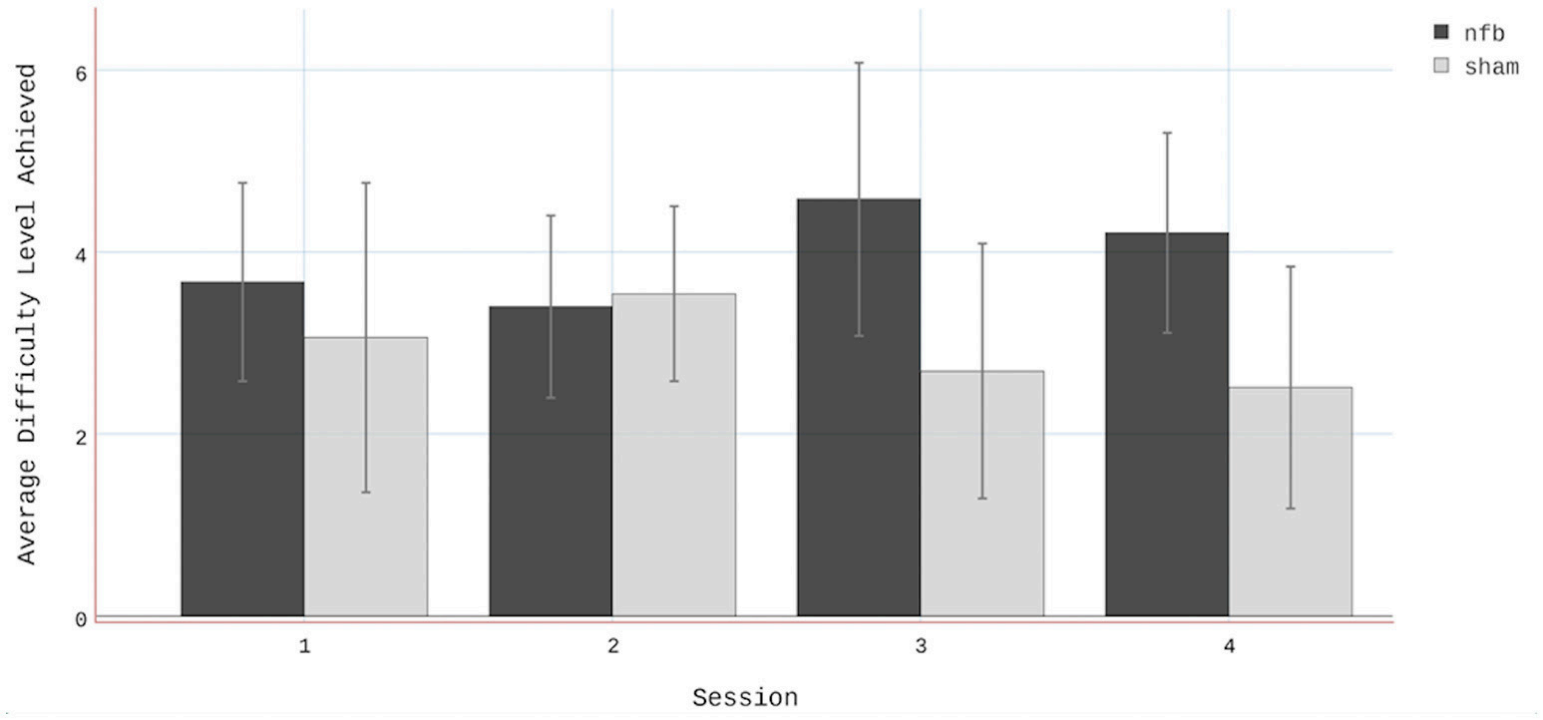
**Average difficulty level achieved by subjects in both the NFB and Sham groups**. Note that there were two difficulty levels below 1 (i.e., 0 and−1). Error bars represent standard deviations.

Finally, the average difficulty level achieved by subjects in the NFB and sham groups for all 4 sessions (Figure 8) reveal a pattern similar to that of the EEG data in the contralateral hemisphere (Figure 4A). The finding that subjects in the NFB group produced greater contralateral sensorimotor activity in sessions 3–4 (in both the NFB and MI task) is mirrored by the finding that these same subjects achieved the highest average difficulty levels in sessions 3–4. The notable exception to this is the fact that the NFB group achieved a higher difficulty level than the sham group during session 1, despite producing significantly less contralateral sensorimotor activity during session 1.

## Discussion

The present work sought to determine the effectiveness of II-NFB to allow subjects an enhanced ability to modulate sensorimotor activity during II. Our analysis rendered mixed results. In the contralateral hemisphere, for II-NFB the effect of session represented the first split in the decision tree (i.e., the split that produced the largest effect), indicating that the effect of session was the largest driver of change. The effect of group within these partitions showed that while in session 1 the NFB group showed higher log2 ERD/S values than the sham group, in sessions 2–4 the NFB group had lower log2 ERD/S values (i.e., more sensorimotor activity). While the superseding effect of session obviously limits our ability to make claims about the effect of our NFB intervention, the fact that the NFB group outperformed the sham group in all sessions after session 1 suggests this group may have benefitted from the NFB training in the sessions following session 1. Furthermore, the analogous findings in the MI task in the contralateral hemisphere further suggest that the NFB training may have influenced MI performance.

Conversely, the findings in the ipsilateral hemisphere—both in the NFB and MI tasks—were the opposite of our hypotheses, with the NFB group showing lower log2 mu ERD values than the sham group. The reason for this finding remains a matter of speculation. One possibility is that our approach to difficulty titration resulted in subjects not having enough exposure to difficulty levels where ipsilateral down-regulation was being factored into the NFB metric calculation, and that in lieu of promoting sensorimotor lateralization, our system primarily promoted a bilateral pattern of sensorimotor activation. This explanation is supported by the fact that the highest average difficulty level for any group-session combination was <5. At levels <5, ERD/S data from the ipsilateral (right) hemisphere was not factored into the NFB Score (Table 1).

Given the fact that subjects in the NFB group were exposed to markedly more NFB where ipsilateral activity was not meaningfully factored into the NFB metric (discussed in detail below), our interpretation of these results are that the individuals in the NFB group were learning to upregulate sensorimotor activity bilaterally over the course of NFB training. This interpretation is consistent with previous research showing a degree of bilateral sensorimotor activation even in unilateral tasks (Kuhlman, 1978; Aziz-Zadeh et al., 2002; Stinear et al., 2006; Kobayashi et al., 2009; Berends et al., 2013; Zimerman et al., 2014), and suggests that ipsilateral signals should have been incorporated into the NFB signal throughout all difficulty levels. Given that other studies have shown ipsilateral down-regulation and contralateral up-regulation when subjects perform NFB incentivizing both of these modulations simultaneously (Boe et al., 2014; Zich et al., 2015), it is possible that we would have seen similar results if we had used a NFB metric that weighted both hemispheres equally throughout the NFB training. The use of a dynamic (and thus non-standardized) learning protocol in the present study, where the difficulty levels of each subject for each session differing based on performance, was adopted given that matching an intervention to one's ability is best practice in learning and rehabilitation contexts (Rebeiro and Polgar, 1999; Page et al., 2013). However, it is possible that its particular implementation in the present study prevented the finding of a more robust effect for our NFB intervention.

However, it is also possible that the attentional demands of II-NFB are such that down-regulating ipsilateral sensorimotor activity during II-NFB is too difficult; it is also possible that four sessions simply did not provide enough training time in order to see the hypothesized effect. This explanation is consistent with past NFB studies showing that >4 sessions are required before individuals are able to gain control over a NFB system (Ros et al., 2010; Gruzelier et al., 2014; Auer et al., 2015).

This shortcoming is not unprecedented in NFB studies involving lateralization of sensorimotor activity. Several other studies attempting to utilize MI-NFB to show that healthy controls could lateralize sensorimotor brain activity found that subjects were successfully able to up-regulate contralateral sensorimotor activity, though they were not able to down-regulate ipsilateral sensorimotor activity (Chiew et al., 2012; Auer et al., 2015).

Furthermore, and again contrary to our expectations, none of the group-session permutations in the NFB task contained a mean log2 ERD/S value of <0 (indicating a decrease in power from baseline). This is an unexpected result given previous findings that typically show a reduction in mu rhythm magnitude during tasks involving sensorimotor processes (Nedelko et al., 2012; Villiger et al., 2013; Kondo et al., 2015). Several studies of II have found it to be associated with increased motor cortex excitability (Sakamoto et al., 2009; Ohno et al., 2011; Tsukazaki et al., 2012; Wright et al., 2014), and increased activation of the motor network more broadly (Nedelko et al., 2012; Villiger et al., 2013), when compared to MI or AO alone. One explanation for the lack of ERD/S values <0 is that engaging with the present NFB system while performing II was too cognitively demanding, hindering subjects' performance of II. Another explanation is that our choice of rest block was not optimal. The choice to have subjects count backwards by 3 s was selected to ensure homogenous activation pattern across subjects and sessions (compared with traditional resting state, where individuals are told to “relax,” and essentially permitted to let their mind wander). However, it is possible that this more-demanding task produced changes in mu power that affected our calculation of ERD/S (i.e., attentional modulation of the somatosensory mu rhythm). Another possible explanation that no mean log2 ERD/S values below zero were seen in the NFB task was related to the choice of motor task included in the video. It is possible that the unusual and complex arm movements contained in the handshake made performing II more difficult, and contributed to our underwhelming results in the NFB task. The decision to use a complex action was to replicate the complex, multi-joint tasks that patients endeavor to recovery in rehabilitation, and the choice of a unique handshake was to ensure all subjects were naïve to the stimulus used.

And lastly, it is possible that the long trial length (50 s) washed out the ERD that was present during the II-NFB task. The majority of studies investigating motor simulation utilize short (<10 s), discrete blocks of MI, AO, or II, and thus it is possible that the difficulty inherent in producing ERD for such an extended period lead to the higher log2 mu ERD/S values we see in the NFB task (see Figures 4A,B). Indeed, the only other NFB system that utilized II had subjects perform 4 s blocks of II (Kondo et al., 2015). While this is a limitation of the current study, clinical studies have shown that MI-NFB, used in conjunction with traditional physiotherapy, can improve patient's clinical outcomes, despite the fact that the patient's ability to gain control of the NFB system was not statistically significant (Ramos-Murguialday et al., 2013; Pichiorri et al., 2015).

Despite these limitations, the present study possesses several noteworthy methodological strengths. The use of an active control, where the NFB and sham groups were exposed to indistinguishable audio-visual stimuli, is critical in order to equate task motivation and interactivity between groups, buttressing our claim that it is NFB itself that is the causal factor responsible for any between-group differences. Furthermore, the design of the NFB system was undertaken with an eye on the potential to translate any possible findings to a real-world setting. Thus the present study endeavored to marry NFB mechanics and interface design with carefully considered user experience (UX) elements (e.g., attempting to reduce frustration through gradual onboarding, striving to drive user motivation through titrating difficulty and simple feedback regarding the users overall progress, and utilizing a novel feedback modality). These design elements were included in the hope that they would make the NFB system more user-friendly, and thus enhance NFB learning. While more simplistic NFB system designs offer more experimental control, given the large upper bound on the potential distribution of NFB interfaces (Kranczioch et al., 2014; Zich et al., 2014), we believe that detailing NFB system design that adopts a more user-centric mentality is a worthy pursuit. However, it appears that the present study's pursuit of this goal impacted the ability to observe a robust NFB training related effect. Our hope is that designers of NFB systems build upon these findings, incorporating complex tasks and titration of difficulty, albeit in a more conservative manner.

Overall, the design features of this II-NFB system, including the use of a complex task performed continuously over a longer window in lieu of a discrete simple one, the titration of difficulty on an individual subject basis, introducing an aspect of gamification by allowing subjects to track their performance during rest periods, and of using a novel feedback modality, were all chosen to enhance UX. While it is the opinion of the authors that NFB systems are too often presented as technical entities that focus on calculation of the NFB signal, with little thought paid to the way that the NFB system design will affect the experience of the user, it is clear that there is a need to balance design features intended to improve UX with more traditional aspects of NFB system design. While it is more laborious, such innovations likely need to be introduced in a piecemeal manner, with a new experimental group for each new UX feature.

The system tested herein represents a novel attempt to uncover the optimal NFB permutation to enhance neurorehabilitation through motor simulation. While it is clear that more research is needed to substantiate the ability of II-NFB to lateralize sensorimotor activity, we hope the methods, results and related interpretation will both inform and inspire future UX-focused NFB experiments.

## Ethics statement

IWK Health Centre-Research Ethics Board (REB). Participants were provided with a consent form fully detailing the rationale behind the study, the time required for the study as well as their compensation. The consent form also outlined the experimental timeline, including a detailed explanation of all procedures and methods involved in the study, including risk. Participants were provided a minimum of 24 h to review the manuscript, and to have any questions regarding their participation answered after which time they signed the form to demonstrate informed consent. There were no additional considerations in the present study.

## Author contributions

CF created the protocol, ran the study, performed data analysis and was the primary author of the manuscript. SB oversaw the study, and was involved in the ideation of the protocol, assisted with experimental design and data analysis, as well as contributed to and oversaw editing of the manuscript. TB assisted with experimental design and data analysis, in addition to editing the manuscript. HN assisted with experimental design, in addition to editing the manuscript.

## Funding

Discovery Grant awarded to SB from the Natural Sciences and Engineering Research Council. Canada Graduate Scholarship awarded to CF from the Natural Sciences and Engineering Research Council.

### Conflict of interest statement

The authors declare that the research was conducted in the absence of any commercial or financial relationships that could be construed as a potential conflict of interest.

## Supplementary material

Supplementary Figure 1 **Depiction of the screen shown to participants during rest blocks in the II-NFB task**. Y-axis depicts difficulty level, X-axis depicts task block.

Supplementary Figure 2 **Decision tree for the CForest predictive model of the ipsilateral (right) EEG sensors during the MI task**.

Supplemental Video 1 **Video depicts the handshake used in the II-NFB task (note that the II-NFB system displayed this video at 6 different levels of colour, from black-and-white to full colour)**.
