# Supplementary figures and images for: Combined Action Observation and Motor Imagery Neurofeedback for Modulation of Brain Activity
Source: Front Hum Neurosci. 2017 Jan 10;10:692. doi: 10.3389/fnhum.2016.00692 (PMC5223402; doi:10.3389/fnhum.2016.00692)

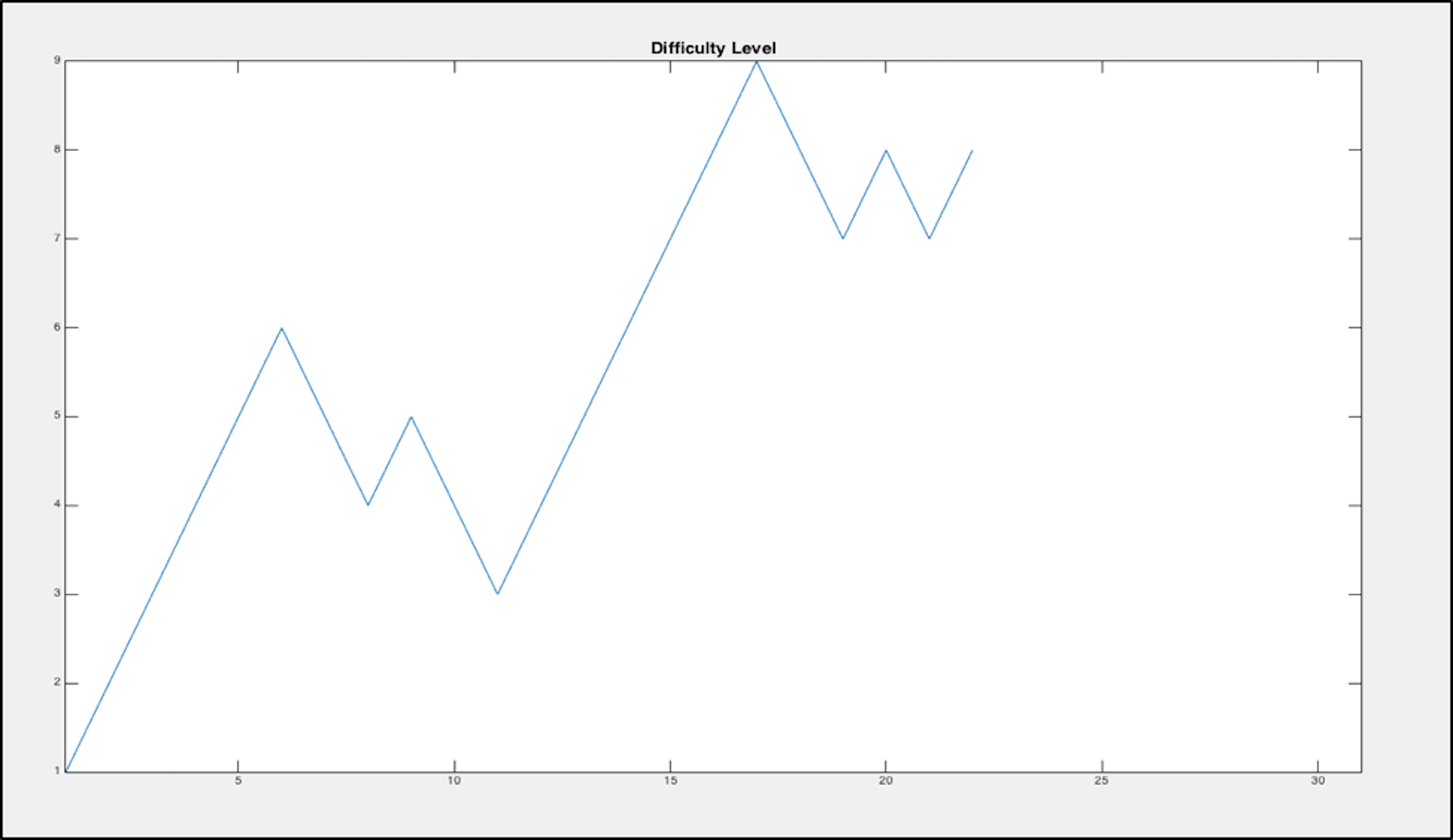

Supplement: Supplementary Figure 1 — Depiction of the screen shown to participants during rest blocks in the II-NFB task. Y-axis depicts difficulty level, X-axis depicts task block. [file Image1.TIFF]

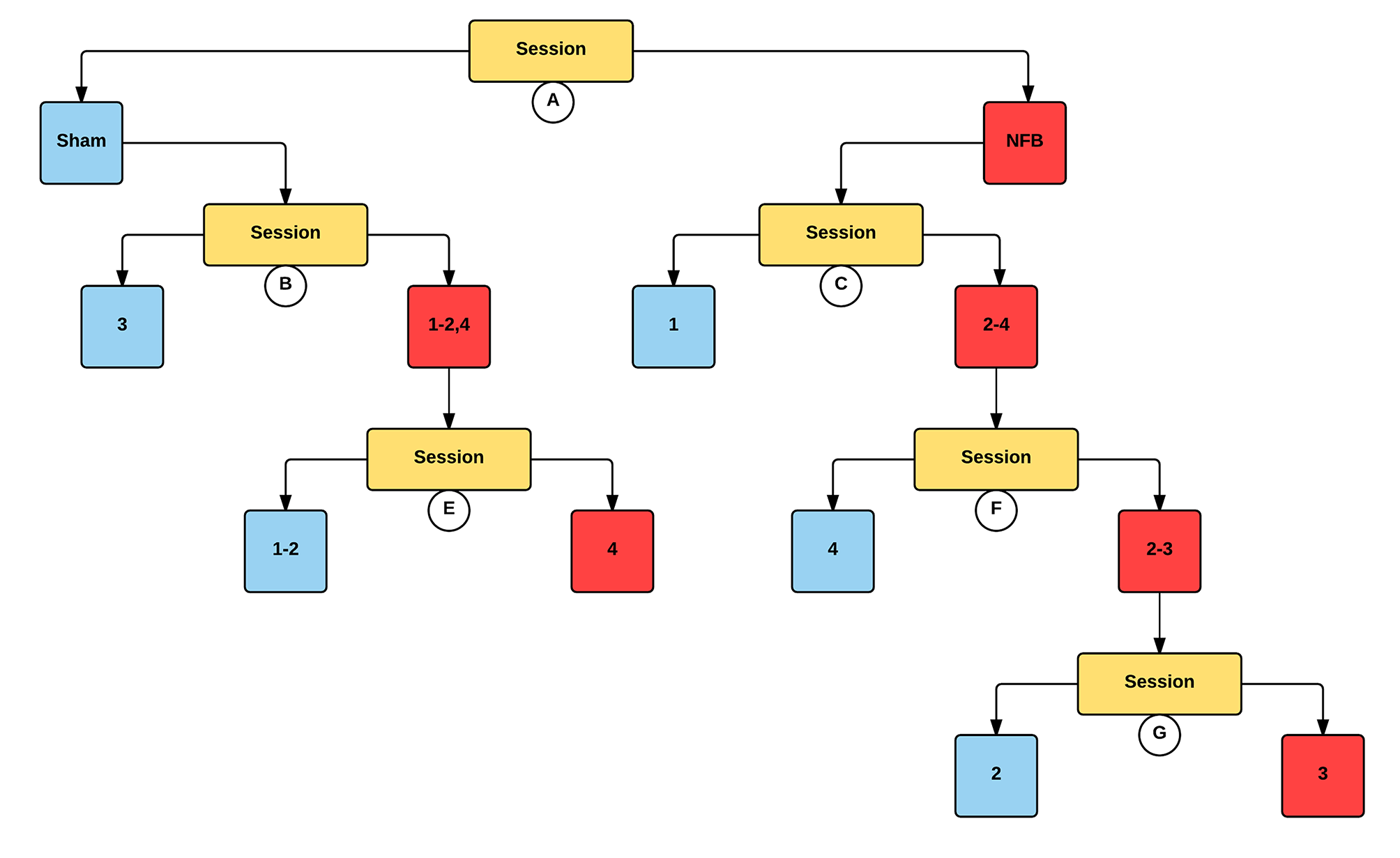

Supplement: Supplementary Figure 2 — Decision tree for the CForest predictive model of the ipsilateral (right) EEG sensors during the MI task. [file Image2.TIFF]
